# Retraction: Downregulated microRNA-30b-3p inhibits proliferation, invasion and migration of glioma cells via inactivation of the AKT signaling pathway by upregulating RECK

**DOI:** 10.1042/BSR-2018-2226_RET

**Published:** 2024-04-29

**Keywords:** AKT signaling pathway, Glioma, MicroRNA-30b-3p, Reversion-inducing cysteine-rich protein with kazal motifs

This article is being retracted from *Bioscience Reports* at the request of the Editor-in-Chief and the Editorial Board following receipt of a notification from a reader, alerting the Editorial Board to concerns surrounding the appearance of Western blots in this paper. The Western blots appear to have minimal background details and share similarities with other Western blots present in papers by different authors. Additionally, the Editorial Team identified undeclared image replacements in Figure 2B, Figure 4A, and Figure 5B, that were introduced during the revision process of this article. The authors have been contacted with regards to the retraction and have not responded to the Journal's queries or the concerns raised. Given the extent of the issues raised, the Editorial Board stand by the decision to retract the article.

